# Survival and Detection of Bivalve Transmissible Neoplasia from the Soft-Shell Clam *Mya arenaria* (MarBTN) in Seawater

**DOI:** 10.3390/pathogens11030283

**Published:** 2022-02-23

**Authors:** Rachael M. Giersch, Samuel F. M. Hart, Satyatejas G. Reddy, Marisa A. Yonemitsu, María J. Orellana Rosales, Madelyn Korn, Brook M. Geleta, Peter D. Countway, José A. Fernández Robledo, Michael J. Metzger

**Affiliations:** 1 Pacific Northwest Research Institute, Seattle, WA 98122, USA; rgiersch@pnri.org (R.M.G.); sfhart@uw.edu (S.F.M.H.); myonemi@uw.edu (M.A.Y.); mkorn1@tulane.edu (M.K.); bgeleta@macalester.edu (B.M.G.); 2 Molecular and Cellular Biology Program, University of Washington, Seattle, WA 98195, USA; 3 Bigelow Laboratory for Ocean Sciences, East Boothbay, ME 04544, USA; sgr87784@uga.edu (S.G.R.); mariajorellanarosale@smccme.edu (M.J.O.R.); pcountway@bigelow.org (P.D.C.); jfernandez-robledo@bigelow.org (J.A.F.R.); 4 University of Georgia, Athens, GA 30602, USA; 5 Southern Maine Community College, South Portland, ME 04106, USA; 6 Tulane University, New Orleans, LA 70118, USA; 7 Macalester College, Saint Paul, MN 55105, USA

**Keywords:** transmissible cancer, contagious cancer, bivalve transmissible neoplasia, BTN, MarBTN, soft-shell clam, *Mya arenaria*, disseminated neoplasia

## Abstract

Many pathogens can cause cancer, but cancer itself does not normally act as an infectious agent. However, transmissible cancers have been found in a few cases in nature: in Tasmanian devils, dogs, and several bivalve species. The transmissible cancers in dogs and devils are known to spread through direct physical contact, but the exact route of transmission of bivalve transmissible neoplasia (BTN) has not yet been confirmed. It has been hypothesized that cancer cells from bivalves could be released by diseased animals and spread through the water column to infect/engraft into other animals. To test the feasibility of this proposed mechanism of transmission, we tested the ability of BTN cells from the soft-shell clam (*Mya arenaria* BTN, or MarBTN) to survive in artificial seawater. We found that MarBTN cells are highly sensitive to salinity, with acute toxicity at salinity levels lower than those found in the native marine environment. BTN cells also survive longer at lower temperatures, with 50% of cells surviving greater than 12 days in seawater at 10 °C, and more than 19 days at 4 °C. With one clam donor, living cells were observed for more than eight weeks at 4 °C. We also used qPCR of environmental DNA (eDNA) to detect the presence of MarBTN-specific DNA in the environment. We observed release of MarBTN-specific DNA into the water of laboratory aquaria containing highly MarBTN-diseased clams, and we detected MarBTN-specific DNA in seawater samples collected from MarBTN-endemic areas in Maine, although the copy numbers detected in environmental samples were much lower than those found in aquaria. Overall, these data show that MarBTN cells can survive well in seawater, and they are released into the water by diseased animals. These findings support the hypothesis that BTN is spread from animal-to-animal by free cells through seawater.

## 1. Introduction

Most cancer stays with the organism from which it came, arising and dying within a single host, but in a few cases, cancer has evolved to transmit from one animal to the next, acting as a pathogen as well as a cancer. The first naturally transmissible cancers to be found were the canine transmissible venereal tumor (CTVT) [1,2] and the Tasmanian devil facial tumor disease (DFTD) [3,4]. More recently, a leukemia-like disease in multiple bivalve species, called disseminated neoplasia (DN) or hemic neoplasia, was shown to be a transmissible cancer in several bivalve species [5]. DN is characterized by massive proliferation of non-adherent, rounded, polyploid neoplastic cells primarily found in the hemolymph of infected bivalves, which disseminate into tissues during later stages of this typically fatal disease [6,7]. DN had been reported for many decades, although the etiology was unknown. Retroviruses or pollution had been thought to be the likely causes [8,9], although two early reports had suggested that it could be due to infectious spread of cancer cells (Sunila et al. 1989 and James Moore’s 1993 Ph.D. Dissertation [10,11]). DN was first confirmed to be a transmissible cancer in soft-shell clams (*Mya arenaria*) [5], and later, multiple independent lineages of transmissible cancer were identified in bivalve species worldwide [12]. To date, seven lineages of the bivalve transmissible neoplasia (BTN) in eight bivalve species have been reported [5,12,13,14,15,16,17], showing that the majority of DN cases in bivalves are likely to be a transmissible cancer, although a few cases of primary DN have been reported [12,14,15]. Given the rapid discovery of BTN lineages (seven lineages identified since 2015) and the fact that more than 20 bivalve species are known to be affected by DN [6,7], many more BTN lineages are likely to be found. There has even been some attempt to use modeling to predict how many exist [18]. In many cases, the BTN that circulates in each species has arisen from a member of that same species. However, increasing cases of cross-species transmission have been reported (one lineage from *Venerupis corrugata* to *Polititapes aureus* in Galicia [12], one from *Chamelea gallina* to *Venus verrucosa* in Europe [17], and a single lineage from *Mytilus trossulus* now found in four *Mytilus* species worldwide [14,15,16]). Furthermore, in some cases, multiple independently derived lineages of BTN have been identified within the same species (as in *Cerastoderma edule* and *Mytilus trossulus*). In soft-shell clams, all analyzed samples of DN since the confirmation of horizontal BTN transmission have been shown to be derived from a single lineage of BTN that arose from one founder clam and has since spread throughout populations along the North American East Coast between Prince Edward Island (Canada) and New York (USA). DN has been reported in *M. arenaria* as early as 1978 [19,20] and it has been observed as far south as Chesapeake Bay, Maryland, USA [9,21,22]. While it is likely that these earlier reports of DN in soft-shell clams represent the same BTN lineage observed today, this cannot yet be confirmed.

CTVT and DFTD are well known to be transmitted by close physical contact, during sex and biting, respectively [1,3,23,24,25], but most adult bivalves are sessile or with limited mobility, without direct contact of soft body tissues, making widespread BTN transmission through direct contact unlikely. Since genomic evidence clearly shows that BTN cells are transmissible [5,12,13,14,15,16,17], they must be able to transmit through a different mechanism than direct physical contact. The most likely route of transmission is the release of the BTN cells into the seawater and uptake of the released cells by naïve animals through filter feeding [5]. For this to occur, BTN cells need to be released from diseased animals and survive in seawater long enough to engraft into a naïve individual. 

A previous report (the first known to propose a transmissible cancer hypothesis for DN in bivalves) characterized the survival of cancer cells from soft-shell clams collected from Chesapeake Bay in 1989. They showed that the cells survive well for at least 6 h in common marine conditions and that survival can be affected by both salinity and temperature [10]. As in Sunila et al., we aim to analyze the ability of BTN cells from soft-shell clams (*Mya arenaria* BTN, or MarBTN) to survive in seawater. We tested the hypothesis that salinity, pH, and temperature affect the survival of MarBTN cells in seawater and extended the length of our study to determine how long the cells can survive at varying temperatures. 

To test whether BTN cells are released from diseased animals into the environment, we developed an environmental DNA (eDNA) qPCR assay. eDNA is DNA collected from environmental samples (e.g., soil, water, snow) rather than from individual organisms. The detection and characterization of eDNA is increasingly finding applications, including detecting bivalve spawning events [26], demonstrating ecological relationships [27,28], and identifying pathogens [29,30]. We tested whether release of MarBTN DNA can be detected in seawater through the analysis of eDNA samples collected from both laboratory and field settings. 

Our findings provide evidence supporting seawater-based transmission of BTN in the wild and provide a proof of principle for the use of eDNA to non-invasively detect and monitor BTN in wild bivalve populations.

## 2. Results

### 2.1. Survival of MarBTN Cells in Seawater and the Effect of Environmental Variables

**Figure 1:**
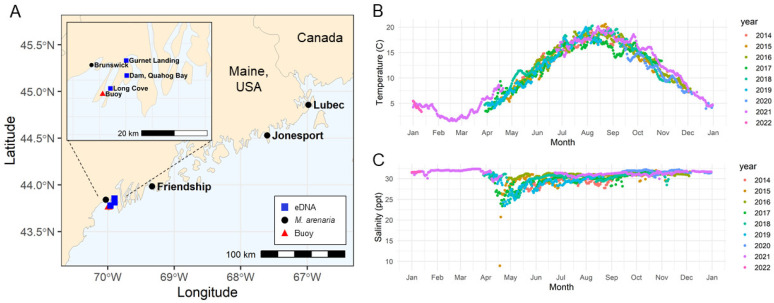
Sampling locations in Maine and seasonal temperature and salinity variation of seawater. (**A**) Map shows the locations in Maine of soft-shell clam (*M. arenaria*) collections (black circles), eDNA sampling (blue squares), and the buoy that provided temperature and salinity data for the region (red triangle), made with the R package ‘rnaturalearthhires’. Data show average of daily readings for (**B**) temperature and (**C**) salinity for the years 2014–2022 from Harpswell Sound, Maine. Environmental parameters were obtained from Bowdoin College’s Land/Ocean Biogeochemical Observatory (LOBO) buoys deployed in Harpswell Sound.

In order to determine the factors that affect survival of *M. arenaria* BTN cells in seawater, we collected MarBTN cells from heavily neoplastic animals from Maine (Figure 1A) and incubated those cells in artificial seawater (ASW) with varying salinity, pH, and temperature, including conditions expected in coastal seawater of the region (Figure 1B,C, and [31]). Identification of dead cells is challenging for marine organisms, as trypan blue (a vital exclusion dye routinely used to discriminate live and dead cells in mammalian cell culture) precipitates out of solution when prepared at salinities found in seawater. Therefore, we tested alternate vital dyes, and found that erythrosine B remains in solution at salinities up to at least twice normal marine salinity (72 g/L Instant Ocean, 59.4 ppt). We verified that the dye is excluded from live cells and properly marks dead cells (Figure S1). 

**Figure 2:**
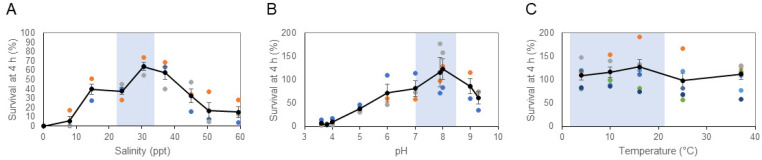
The effect of salinity, pH, and temperature on survival of MarBTN cells in artificial seawater. BTN cells from soft-shell clams (*M. arenaria*) were collected and incubated in ASW with varying (**A**) salinity, (**B**) pH, and (**C**) temperature for 4 h before survival was measured, using erythrosine B to identify viable cells. Unless it was the variable being tested, ASW was prepared at normal marine salinity (30.5 ppt), without additional pH modification (pH 7.93), and held at 16 ℃. For pH and temperature experiments, live cell counts at 4 h were normalized by cell counts for each well at initiation of the experiment, but due to acute toxicity of low salinity, this could not be done for part A. These counts were normalized by the expected number of cells based on cell counting of the initial cell suspension, assuming no loss during centrifugation and pipetting. For each experiment, we used MarBTN cells from 3–6 separate diseased clam donors (colored points, with each color representing cells from a different clam donor). The average is shown (black points with line) and error bars show the standard error of the mean. Light blue shading indicates approximate range expected in coastal Maine seawater (see Figure 1 and pH data from Casco Bay [31]). Specific identity of clam donors is listed in Table S1.

As found in the previous study of bivalve DN cells, MarBTN cells rapidly die in low salinity water (Figure 2A). In contrast, the majority of cells survive at least 4 h in ASW of consistent with marine salinity in the New England area (30.5 ppt). Significantly decreased survival was observed at ≤23.9 ppt and ≥45 ppt (*p* < 0.05). MarBTN cells also show the greatest survival at expected marine pH, but significant cell death only occurred in highly acidic conditions not likely to be relevant to the environment (Figure 2B, *p* < 0.05 only for pH 4.0 and lower). Variation of temperature from 4 to 37 °C had minimal effect on survival within 4 h (Figure 2C, *p* > 0.05 for all conditions). 

**Figure 3:**
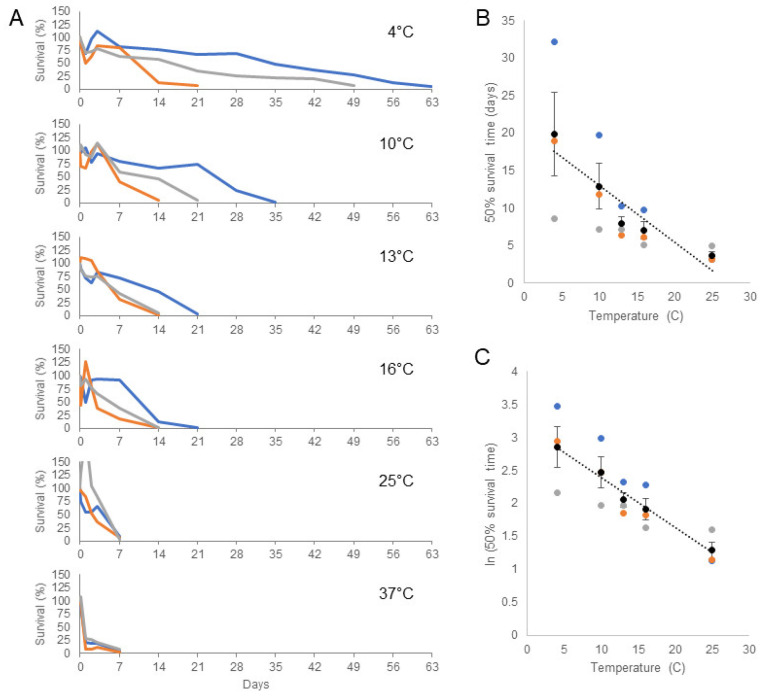
The effect of temperature on long-term survival of MarBTN cells in artificial seawater. MarBTN cells were collected from three different diseased clams and incubated in ASW (30.5 ppt, pH 7.93, with penicillin / streptomycin / voriconazole). Cells were incubated in 4, 10, 13, 16, 25, and 37 ℃. (**A**) Cell survival was monitored by resuspension and removal of an aliquot of cells, counted using erythrosine B, at 4 h, 1 d, 2 d, 3 d, 1 wk, and weekly beyond that. Experiments were stopped when survival dropped below 10%. Each color represents a separate biological replicate of cells from a different clam donor (ID in Table S1). A single outlying value of 206% for FFM-26D8 (grey) at 25 ℃ at 24 h is off the scale as shown. (**B**) 50% survival time was estimated using linear regression of each biological replicate and was plotted against temperature (excluding 37 °C), and (**C**) natural log transformed survival time was also plotted. Black dots are the average and error bars show standard error of the mean. The dotted line is a linear regression of the average values. Both models suggest that there is an association between lower temperature and longer survival times (*p* < 0.05 and R^2^ = 0.8754 for B, and *p* < 0.01 and R^2^ = 0.9845 for C).

A four-hour incubation was chosen for the initial survival experiments as we found that proliferation of bacteria, protists, and unknown ciliates led to inconsistent cell survival in ASW beyond short-term incubation (this same problem also prevented Sunila et al. from measuring survival beyond 6 h). We recently found, however, that with the use of penicillin/streptomycin and, notably, the addition of voriconazole, contaminant overgrowth could be controlled. We were therefore able to follow MarBTN cell survival long-term at varying temperatures. We found that cells were able to survive far longer than four hours in 1× ASW approximating typical marine conditions (Figure 3A). We observed some variability in survival times for cells from different donor animals, but overall, we found that cells consistently survived longer at colder temperatures. At 10 °C, 50% of cells survived 12.9 days, and at 4 °C, 50% survival reached 19.9 days. For cells from one animal, >50% of cells were still alive after one month at 4 °C, and living cells could still be detected after more than 8 weeks. 50% survival was 8.0, 7.0, 3.7, and 1.6 days at 13, 16, 25, and 37 °C, respectively, demonstrating a significant association between lower temperature and longer survival (Figure 3B,C). The cause of the marked variability observed in long-term survival between BTN cells from different donors is unknown. This could be due to differences in the stage of disease progression, different genotypes of the host, or differences in the subclone of the BTN itself. Regardless, this dramatically increases the amount of time BTN cells are known to survive in ASW, showing that—at least for the MarBTN lineage—these transmissible cells likely survive long enough to broadly disseminate through seawater to infect other clams. 

### 2.2. Detection of MarBTN eDNA Released from Diseased Clams in Laboratory Aquaria 

**Figure 4:**
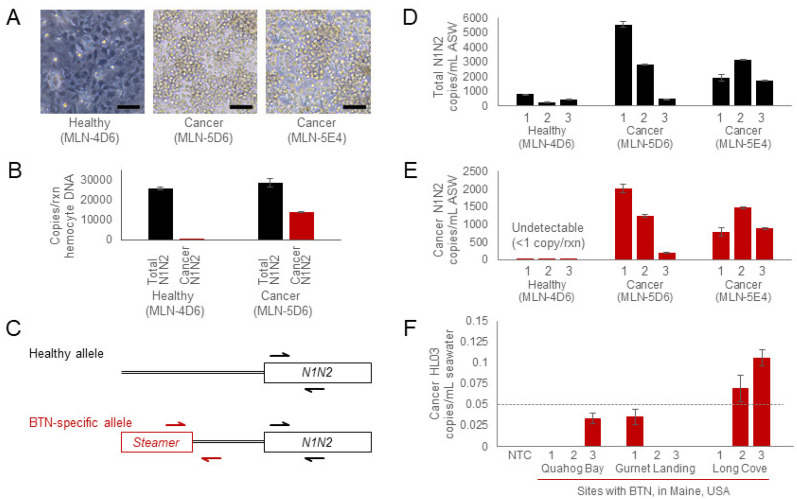
Detection of MarBTN eDNA from seawater in aquaria and from sites of known endemic MarBTN. Representative healthy and cancerous soft-shell clams were identified (**A**) through a screen of hemocyte morphology (scale bar is 50 µm), and (**B**) the diagnosis was confirmed using a qPCR analysis of genomic DNA from hemocytes obtained from one of the diseased animals and the healthy animal used in the subsequent eDNA experiment. (**C**) The schematic shows the healthy *N1N2* allele and the cancer-associated allele, with arrows indicating the locations of the control primers (Total *N1N2*, black), used to determine the total number of clam alleles, and primers specific for the MarBTN lineage (Cancer-*N1N2*, red), used to quantify MarBTN DNA. For eDNA analysis, each animal was housed in a separate aquarium, and eDNA was extracted from aquaria water on 3 sequential days. qPCR was used to quantify (**D**) the total copies of *N1N2* alleles and (**E**) copies of the cancer-associated-N1N2 allele using the primer pairs diagrammed in C. (**F**) Samples of water from sites in Maine with soft-shell clams known to have BTN were collected and eDNA was extracted. For each site, one water sample was collected, and three sub-samples were extracted separately (one bar for each sub-sample). qPCR analysis of the MarBTN-specific marker (Cancer-HL03) confirms detection of BTN DNA in the water. The dotted line shows 1 copy/reaction based on the plasmid standard curve. For all qPCR, each sample was run in three reactions, and the values presented are averages of the triplicate results, with standard error of the mean of these technical triplicate wells shown as error bars. The average value was shown to be above zero only if the product was detectible in all triplicate reactions. Water was used as the no-template-control and was undetectable in all three wells. Copy numbers per µL DNA were converted to copies/mL, based on normalization to the total volume of water extracted.

For BTN to be spread through the water, cells need to survive, but they also need to get out into the water from within a diseased animal. To test whether MarBTN cells are released by diseased clams, we developed a qPCR assay to identify markers found only in MarBTN cells and not in healthy *M. arenaria* genomes. qPCR analysis of hemocyte genomic DNA from healthy and heavily diseased animals confirms that DNA from healthy cells amplify only with the healthy *N1N2* control primers, while MarBTN cell DNA amplified with both the normal and the cancer-specific primer pairs, and the ratio of the amplification shows that the cancer-associated allele is heterozygous in MarBTN (Figure 4A–C). 

To test whether MarBTN-specific DNA is released and can be subsequently detected in seawater, we housed individual healthy and highly neoplastic animals in separate aquaria and collected eDNA from the water for three consecutive days (Figure 4D,E). The qPCR data confirm that the healthy animal releases some normal DNA and no detectible MarBTN-specific DNA, while both heavily diseased animals release significant amounts of cancer-specific DNA consistently on all three days, although the amount does vary from one 24-hour period to the next. This pattern was confirmed using the secondary HL03 cancer-specific primer set (Figure S2). Additionally, the ratio of cancer-*N1N2* to total-*N1N2* ranges from 0.41–0.51, showing that ≥82% of DNA in the water from both diseased animals came from released MarBTN cells (the cancer cells contain both alleles with the insertion and without, so pure MarBTN cells have a 0.5 cancer-allele fraction). This suggests that diseased animals are expelling far more MarBTN cells than healthy host cells.

### 2.3. Detection of MarBTN in Natural Sites of Known Endemic BTN

The natural clam environment is far larger than a 1 L tank, so we next tested whether MarBTN-specific DNA could be detected in wild environments where clam populations are known to be affected by endemic MarBTN. Even after cell death, MarBTN-specific DNA can be stable in ASW for many days—we observed a trend toward decreased MarBTN amplification, but no statistically significant difference, when killed MarBTN cells were incubated in ASW at 4 °C for up to a week (Figure S3). Therefore, eDNA can be both a highly sensitive and specific assay, able to detect DNA from MarBTN cells in the environment, even if it cannot specifically determine whether the DNA was from cells living at the time of collection. 

We chose three populations in Maine, collected surface water samples from each site, extracted eDNA, and performed qPCR (Figure 4F). These results show MarBTN-specific DNA at copy numbers far lower than those found in aquaria with heavily diseased animals, as expected, but we did observe MarBTN-specific amplification in two of the three sub-samples from Long Cove at a level above 1 copy/reaction (with amplification observed in all triplicate reactions for those two eDNA subsamples). These results demonstrate that MarBTN-specific DNA can be found in field samples of seawater in addition to being found in more concentrated laboratory conditions, again providing evidence for the hypothesized seawater-transmission of BTN.

## 3. Discussion

This study has shown that MarBTN cells can survive for many weeks in seawater under the right conditions, that they are acutely impacted by salinity, but not pH, and that they can survive longer at colder environmental temperatures. We also show that eDNA from MarBTN cells can be detected in both aquaria and field samples, providing evidence for release of BTN cells from diseased animals for the first time. The previously proposed mechanism of transmission of BTN through seawater requires both long-term cell survival in the environment and release of BTN cells into the environment by diseased animals. This study provides evidence supporting both of those requirements. 

This study largely agrees with the findings of Sunila et al. [10], showing the strong effect of salinity, but minimal effect of pH, and minimal effect of temperature on short-term survival (except for toxicity at high temperatures). However, the cancer cells in the previous study demonstrated optimal survival in 10–15 ppt, a salinity level that was rapidly lethal to the cells in this study. Notably, the samples from that study were collected from northern Chesapeake Bay. In that estuary environment, the surface seawater was 10 ppt, whereas the samples in the current study were taken from the coast of Maine, where the seawater has a much higher salinity (ranging from 23–33 ppt, Figure 1). Sunila et al. had hypothesized an infectious cause for DN in *M. arenaria*, but it had not been shown at the time of the study that DN in *M. arenaria* was a BTN, so it is unclear whether the cancer cells in that study were from the same MarBTN lineage that is currently affecting New England and Prince Edward Island clams. The differences between these two findings suggest that there may have been evolution of MarBTN to adapt to the specific environment in which its hosts reside and in which it must survive in order to transmit. This could represent evolution of two separate lineages within different environmental conditions, or it could represent divergence of a single lineage to better survive in marine vs. estuarine environments. This should be tested in future studies. Regardless, both studies showed that cancer cells were acutely sensitive to salinities lower than 10 ppt. To date, no DN has been observed in freshwater environments, so low-salinity environments may provide a potential “safe harbor” for bivalves, where transmissible cancer cannot survive or efficiently transmit.

We found clear evidence that MarBTN cells survive longer in the environment in colder temperatures, which may have implications for understanding the seasonality of BTNs. BTN in soft-shell clams and other species have been reported to have seasonal fluctuations in prevalence [21,32,33], with a higher prevalence usually seen in winter months and lower prevalence in summer. The survival data presented here suggest that transmission may be more efficient in colder seasons due to longer environmental survival of released BTN cells, and this may be a factor in the observed higher prevalence in colder months. There are, however, additional unknown factors, such as the effect of temperature on the progression of disease, which may greatly affect seasonality as well and which should be investigated. 

A very recent study of the MtrBTN2 lineage of transmissible cancer, known to infect four *Mytilus* species around the world [14,15,16], has shown that these cancer cells also can survive for a few days in seawater [34]. The authors assayed cell survival at 18 °C, and our results showing longer survival of MarBTN at lower temperatures suggest that their finding of 6-day survival may be an underestimate. It will be interesting to determine in the future whether our finding of the effects of salinity and temperature on environmental cell survival are the same across BTNs from different species and whether variable environmental conditions affect the spread of BTN lineages in different geographic locations around the world. 

The detection of MarBTN DNA released into tank-water of heavily diseased animals provides strong evidence that these parasitic cancer cells are released into the environment, but the mechanism of BTN cell release is unknown. Healthy clams do release some normal clam DNA, but our finding that diseased clams seem to release more cells than normal clams do (and they seem to specifically release MarBTN cells), suggests that this may be an active and specific process. Interestingly, a recent study showed that, under temperature stress conditions, the hemocytes of normal marine mussels can be released into the seawater [35]. It is reasonable to hypothesize that BTN cells in mussels and other bivalves could use this same stress-induced mechanism to be released from animals. Whether the specific trigger for cancer cell release is temperature, progression of the disease, or some other factor remains an important question that will impact our understanding of BTN transmission dynamics.

One limitation of our study is that detection of MarBTN-specific eDNA does not confirm that live cells are in the environment, only that the DNA can be detected. However, given the fact that BTN cells in different animals are identical to each other, and that BTN cells can survive well in the marine environment, it seems reasonable to conclude that eDNA is detecting live cells. This study provides the proof of principle for an eDNA assay that can be used to determine the timing of cell release during BTN progression. It can also be used to identify the presence of BTN in field samples, potentially serving as a non-invasive proxy for monitoring disease in the wild and possibly reducing the requirement for more invasive and expensive screening of animals for disease. eDNA could be used as a cost-effective screening tool for analysis of disease dynamics within a population known to be affected by any known BTN, and it could be used to screen for the invasion of previously naïve populations. At least in the case of the MtrBTN2 lineage in mussels, long-distance transmission is likely due to accidental transport along human shipping routes [14,16], suggesting that surveying of potentially susceptible bivalve populations worldwide could be useful in determining the spread of these parasitic cancers. 

In this study, we show evidence supporting the long-term survival of MarBTN cells and release of MarBTN cells from diseased animals. These cancer cells therefore have the capacity to spread from individual to individual, through the environment, blurring the line between cancer metastasis and a parasitic organism. Overall, these data provide proof of principle supporting the transmission of BTN through the seawater as a pathogen, and they establish new methods to investigate the mechanisms of BTN survival, progression, and spread.

## 4. Materials and Methods

### 4.1. Temperature and Salinity Data Access

The temperature and salinity time series data were obtained from the Land-Ocean Biogeochemical Observatory (LOBO) in Harpswell Sound [36] at 43.761667°, −69.988333° (LOBO-0052, http://bowdoin.loboviz.com/). Data from 14 May 2014 through 11 January 2022 were retrieved (data accessed on 2 February 2022). A daily average was calculated on each day where recordings were made, and data were plotted using ggplot2 [37].

### 4.2. Collection of Clams and MarBTN Cells

Adult soft-shell clams (*M. arenaria*) >50 mm in length were collected by commercial sources from multiple locations in Maine, USA (Table S1), and animals were housed in 1× ASW (36 g/L Instant Ocean, Blacksburg, VA, USA), in aerated aquaria, supplemented 2–3 times weekly with PhytoFeast or LPB Frozen Shellfish Diet (Reed Mariculture, Campbell, CA, USA). Animals were screened for high levels of DN as described below [5]. Approximately 0.5–1 mL of hemolymph was collected from the pericardial sinus of each animal using a 0.5 in 26-gauge needle fitted on a 3 mL syringe. 4–5 drops from the syringe (~50 µL, just enough to cover the bottom of the well) were placed in a well of a 96-well plate and incubated at 4–10 °C for 1 hour to allow the cells to settle. Wells were screened for clams with high levels of MarBTN based on morphological differences between healthy hemocytes and MarBTN cells on an inverted phase-contrast microscope. BTN cells are rounded and refractile and do not adhere to the bottom of the well, while healthy hemocytes adhere tightly to the well and extend multiple pseudopodia. As we were interested in testing the properties of the MarBTN cells and not host cells, only animals with ≥75% of cancer cells in their hemolymph were used in survival experiments. 

### 4.3. Counting Live Cells in Artificial Seawater

We used the alternate vital dye, erythrosine B (MilliporeSigma, Burlington, MA, USA) to stain samples to discriminate live and dead cells during counting. This dye is soluble throughout all salinities used in this study (0–72 g/L Instant Ocean, 0–59.4 ppt, 1–1.045 specific gravity). To count live cells, 10 µL of ASW containing cells were mixed 1:1 with 2× erythrosine B solution (10 µg/mL, dissolved in PBS4, which is phosphate buffered saline (PBS, Genesee Scientific, El Cahon, CA, USA) plus 400 mM NaCl to approximate marine salinity). After 10 min at room temperature, live cells were counted manually on a hemocytometer on an inverted phase-contrast microscope, counting only rounded, refractive cells that exclude dye. 

### 4.4. Validation of Erythrosine B Live/Dead Staining

200 µL of hemolymph was bled from a highly neoplastic animal, FFM-26C10. From the same hemolymph sample, four 50 µL aliquots were treated in four different ways to compare live cell exclusion and dead cell uptake of the stain: (A) Hemolymph; (B) Hemolymph spun down at 500× *g* for 10 min at 4 °C, supernatant removed, and cells resuspended in 1× ASW; (C) Hemolymph frozen at −20 °C overnight to induce cell death, then thawed the next day on ice; (D) Hemolymph spun down at 500× *g* for 10 min at 4 °C, supernatant removed, and cells resuspended in deionized (DI) water for 1 h on ice to induce cell death. For each condition, the cell samples were stained 1:1 with 2× erythrosine B and incubated on ice for 10 min, then imaged using a hemocytometer as described above. 

### 4.5. Short-Term Cell Survival Assays

For all MarBTN cell survival assays, hemolymph was collected from the pericardial sinus area of each heavily neoplastic animal using a 0.5 in 26-gauge needle. To remove host cells, the hemolymph was allowed to sit on a 6 cm tissue culture dish or 24-well plate at 4 °C for one hour. Healthy hemocytes from the host adhered to the dish, and the hemolymph containing the non-adherent MarBTN cells was resuspended and removed from the plate. The non-adherent cell suspensions were then spun down at 500× *g* for 10 min at 4 °C. Hemolymph was removed, and MarBTN cells were resuspended in 1× ASW: filter-sterilized Instant Ocean in DI water with no additives, 36 g/L, 30.5 ppt, and pH 7.93. For salinity, cells were diluted to an approximate concentration of 1 × 10^6^ cells/mL, and 20 µL of cells were added to 180 µL of ASW with varying concentrations of Instant Ocean, from 0 to 2× the normal salinity level (with specific gravity measured by a refractometer at 25 °C, converted to ppt with https://reefapp.net/en/salinity-calculator (accessed on 2 February 2022)), and placed in wells of a 96-well plate at 16 °C. For pH, cells were aliquoted into multiple tubes, spun a second time, and cells in each tube were resuspended in 200 µL of ASW with differing pH (3.8–9.3, modified with 2M NaOH or 3N HCl), with a target of 1 × 10^6^ cell/mL, and put into wells of a 96-well plate at 16 °C. For temperature, the cells were resuspended in 400 µL of 1× ASW (30.5 ppt, pH 7.93) and placed in wells of a 24-well plate at the indicated temperatures (4–37 °C). Cell survival at 4 h was counted with erythrosine B staining as described above. For pH and temperature, cell counts at time zero were used to normalize cell survival. Cell counts at time zero were not possible in the salinity experiment, as cell death in DI water was too rapid, so these counts were normalized to the concentration expected based on cell counts before the final centrifugation and resuspension (assuming no cell loss during centrifugation). Statistical significance was determined through a two-tailed *t* test assuming equal variance, comparing each condition to survival at standard ASW (30.5 ppt, 7.9 pH, 16 °C).

### 4.6. Long-Term Cell Survival Assays

For long-term cell survival, penicillin/streptomycin (1×, GenClone, Genesee Scientific) and voriconazole (1 mM final concentration, Acros Organics, Thermo Fisher Scientific, Waltham, MA, USA) was added to ASW. Other antimicrobial drugs were tested (e.g., moxifloxacin, doxycycline, metronidazole, and triclosan) but were not found to reduce contaminants at a concentration that was non-toxic to MarBTN cells (Table S2). Cells were collected as above and resuspended in 400 µL 1× ASW with penicillin/streptomycin/voriconazole, in wells of a 48-well plate, at 2 × 10^5^–2 × 10^6^ cells/mL. At each timepoint (time 0, 4 h, 1 d, 2 d, 3 d, 7 d, and weekly after that), 10 µL of cells were removed for counting of live cells using erythrosine B, as described above. After each timepoint, the volume was measured by pipette, and ASW with antimicrobial drugs was added to replace the media removed for cell counting and lost due to evaporation. Live cell counts at each timepoint were normalized by live cell counts at time zero to calculate survival, and the normalization value was multiplied by 0.975 after each additional timepoint to reflect the removal of 10 µL of cells from the original 400 µL sample. 50% survival times were estimated by fitting a linear regression for each sample and using the model to calculate 50% survival. *t* and *p* values were then calculated for the linear regression of the average 50% survival times at different temperatures. 

### 4.7. eDNA Extraction from Aquaria

Animals were maintained at approximately 10 °C in individual tanks in 1 L of 1× ASW, with constant aeration. 24 h prior to water collection, the entire volume of the tank water was replaced. Each day for three consecutive days, 250 mL of each water sample was collected after mixing, and the entire tank water was replaced, so that each sampling is from water with 24 h of exposure to a single clam. Water samples were vacuum filtered through a 47 mm 0.45 µm cellulose nitrate filter. Using forceps, the filtered sample/paper was folded small enough to fit into a 2 mL tube and frozen at −80 °C until extraction was performed. 

Extraction protocol was modified from Renshaw et al. [38]. Briefly, 900 µL CTAB buffer (2% CTAB *w*/*v*, 20 mM EDTA, 100 mM Tris-HCl, and 1.4 M NaCl, in water) was added to the filter, and the tubes were incubated at 65 °C for 30 min. Tubes were spun to collect the sample in the bottom of the tube and 900 µL chloroform:isoamyl alcohol (24:1) was added, followed by shaking or vortexing. Tubes were spun for 5 min at 15,000× *g*, and the 700–850 µL aqueous layer was transferred to a new tube with 700 µL chloroform. This was shaken and spun as before and the ~700 µL aqueous layer was transferred to a new tube containing 700 µL cold isopropanol and 24 µL 5M NaCl. 4.67 µL glycogen blue was added to ensure visibility of the pellet, and samples were allowed to precipitate overnight at −20 to −30 °C. DNA was spun for 10 min at 15,000× *g* and the liquid removed by pipette. 500 µL of 75% ethanol was slowly added, the tube was spun a second time, and the ethanol was poured off. DNA pellets were air dried and resuspended in 100 µL Buffer EB (Qiagen, Hilden, Germany).

### 4.8. Field Seawater Collection and Extraction of MarBTN eDNA

Seawater samples were collected from surface water overlying clam-flats in Maine by filling a single 4-liter acid-washed (5% HCl) HDPE bottle from each location (Quahog Bay Dam, June 6, 2021, 43.812541°, −69.896802°; Gurnet Landing, June 6, 2021, 43.853734°, −69.898677°; and Long Cove, June 13, 2021, 43.777156°, −69.958582°). Sample bottles were stored in a cooler with ice packs until delivery to Bigelow Laboratory within 24 h of collection. Triplicate sub-samples of 500 mL seawater were filtered from each bottle onto 47 mm diameter, 0.2 µm Supor filters (Pall Corp., Ann Arbor, MI, USA) to collect environmental DNA. Filters were rolled and placed in 4.5 mL cryovials (USA Scientific, Ocala, FL, USA) for storage at −80 °C until DNA extraction. Filters were rolled to ensure that the particle-bearing filter surface faced inward and that the filter would unfurl when it was transferred to a DNA extraction tube. 

Environmental DNA was extracted from the Supor filters using the DNeasy PowerWater kit (Qiagen). Frozen filters were transferred from the cryovials to the 5 mL PowerWater bead tubes and 1 mL of warmed (55 °C) PW1 solution was added. Bead tubes containing a filter, PW1 solution and garnet beads were vortexed for 30 min on a Vortex Genie IIT (Scientific Industries, Bohemia, NY, USA) using a 15 mL tube adapter. After the bead-beating step, the crude cell lysate, extracted Supor filter, and most of the beads were tapped into the barrel of a sterile 10 mL syringe held over a 2 mL Eppendorf DNA LoBind tube to catch sample lysate. The syringe’s plunger was inserted a short way into the syringe barrel before the syringe assembly was flipped upright to purge air. The syringe assembly was inverted over the 2 mL LoBind tube for a second time, and the remaining lysate was pressed out of the bead and filter slurry. The volume of this crude sample lysate was recorded, and the remainder of the DNA extraction procedure followed the kit protocol. Extracted DNA samples were stored in DNA LoBind tubes at −20 °C until analysis by qPCR.

### 4.9. qPCR of Hemocyte DNA and eDNA 

To quantify the presence of neoplastic DNA in a hemolymph genomic DNA or eDNA sample, allele-specific qPCR was performed using four sets of primers (Table S3). Both cancer-specific primer pairs amplify specific integration sites of the LTR-retrotransposon *Steamer*, found only in MarBTN cells (*Steamer* is highly amplified within *M. arenaria* BTN cells, [39]). The primary locus was a MarBTN-specific insertion of the LTR-retrotransposon *Steamer* at the *N1N2* gene (identified through preliminary analysis of MarBTN genome sequencing). A MarBTN-specific primer pair targeting this insertion junction amplifies half the total amount of *N1N1* alleles in a cancer cell (as the insertion is in two of four copies of the gene in a tetraploid region) and a primer pair in a conserved region of the *N1N2* ORF nearby quantifies the total copies of the *N1N2* locus present. The ratio of the two can be used to determine the fraction of clam hemolymph made up of MarBTN cells. A single plasmid (pCR-SteamerLTR-N1N2) was used for the standard curve. It was made by cloning the *Steamer*-*N1N2* amplicon, amplified from genomic DNA of MarBTN cells (Zero Blunt TOPO PCR cloning kit, Invitrogen, Waltham, MA). The secondary marker was a different MarBTN-specific *Steamer* integration site, termed HL03 [38]. A plasmid was cloned which includes both the HL03 locus and a separate conserved region of the EF1α gene as a control (pIMHL03c2-EF1α). Primers used for cloning control plasmids are listed in Table S3, and sequences have been archived in GenBank (accession numbers OM105837-9). The plasmid concentration was measured (Qubit, Thermo Fisher Scientific) and copy number per µL was calculated based on the plasmid sizes. Plasmids were linearized with 0.25 µL of NotI-HF (NEB, Ipswitch, MA, USA) for 30 min at 37 °C in a 20 µL reaction at 1 × 10^10^ copies/µL, heat-inactivated 20 min at 65 °C, then diluted to 1 × 10^9^ with 180 µL Buffer AE (Qiagen). Standard curves were prepared from 1 × 10^7^ copies/rxn to 1 × 10^1^ copies/rxn. For aquaria samples, 2 µL of extracted eDNA was run in 10 µL reactions on a StepOnePlus real-time PCR cycler (Applied Biosystems, Waltham, MA, USA). For field eDNA samples, 4 µL of eDNA was used in a 20 µL reaction for increased sensitivity. Reactions were run as follows: 95 °C for 2 min, 40 cycles of 95 °C for 15 s and 60 °C for 30 s, followed by a melt curve using 95 °C for 15 s, 60 °C for 1 min, and ramping 0.3 °C from 60 °C to 95 °C, followed by a 15 s hold at 95 °C. All samples were run in triplicate and values presented are an average of triplicates, treating wells with undetectable amplification as zero copies. Due to the potential for contamination with the control plasmid, only the HL03 marker was used for analysis of field samples, as this plasmid was not present in the lab where eDNA was extracted.

## Data Availability

All data used in this paper are provided in the results and supplementary materials.

## Supplementary Materials

The following supporting information can be downloaded at: https://www.mdpi.com/article/10.3390/pathogens11030283/s1, Figure S1: Validation of erythrosine B as a vital dye in seawater; Figure S2: Detection of MarBTN eDNA from seawater in aquaria with secondary qPCR marker; Figure S3: Detection of MarBTN eDNA from dead MarBTN cells; Table S1: Collection information of soft-shell clams (*Mya arenaria*) used in this study; Table S2: List of tested antimicrobial drugs and concentrations; Table S3: Primers used in qPCR and cloning; Table S3: Data for Figure 2A; Table S4: Data for Figure 2B; Table S5: Data for Figure 2C; Table S6: Data for Figure 3A; Table S7: Data for Figure 3B,C; Table S8: Data for Figure 4B and Figure S1A; Table S9: Data for Figure 4D,E and Figure S1C,D; Table S10: Data for Figure 4F.
