# Supplementary material for: Survival and Detection of Bivalve Transmissible Neoplasia from the Soft-Shell Clam Mya arenaria (MarBTN) in Seawater
Source: Pathogens. 2022 Feb 23;11(3):283. doi: 10.3390/pathogens11030283 (PMC8955499; doi:10.3390/pathogens11030283)
Supplement: Supplementary file 1 [file pathogens-11-00283-s001.zip › Table S-2022-02-04.pdf]

Table S1. Collection information of soft-shell clams (*Mya arenaria*) used in this study

Animal ID	Cancer in hemolymph[1]	Arrival date	Harvest location	Figure (color)
FFM-4D7	100%	10/22/2019	Lubec, ME	1A (orange)
FFM-4G8	100%	10/22/2019	Lubec, ME	1A (blue)
FFM-6B8	75%	10/31/2019	Brunswick, ME	1A (grey)
FFM-26C1	100%	10/1/2021	Friendship, ME	1B, 2, S2 (blue)
FFM-26C9	100%	10/7/2021	Friendship, ME	1B & S4 (orange)
FFM-26D8	100%	10/7/2021	Friendship, ME	1B, 2, S2 (grey)
FFM-5D4	75%	10/31/2019	Brunswick, ME	1C (light blue)
FFM-7A12	100%	12/10/2019	Jonesport, ME	1C (green)
FFM-7F4	100%	12/10/2019	Jonesport, ME	1C (dark blue)
FFM-8C3	100%	1/7/2020	Brunswick, ME	1C (orange)
FFM-9A1	100%	1/7/2020	Brunswick, ME	1C (blue)
FFM-9A12	100%	1/7/2020	Brunswick, ME	1C (grey)
FFM-26C10	100%	10/7/2021	Friendship, ME	2 & S2 (orange)<br>S4 (grey), S1
MLN-4D6	0%	1/29/2019	ME	3, S3
MLN-5D6	100%	2/19/2019	ME	3, S3
MLN-5E4	100%	2/19/2019	ME	3, S3
FFM-26C4	100%	10/7/2021	Friendship, ME	S4 (blue)

1 Cancer in hemolymph was first estimated through microscopic analysis of hemolymph. Only highly diseased animals were selected for use as donors for BTN cell survival experiments

Table S2. List of tested antimicrobial drugs and concentrations

Drug	Concentration	Parasite growth	Toxic to BTN
Triclosan	1µM	Y	N
	10 µM	Y	N
	50 µM	Y	Y
	100 µM	Y	Y
Doxycycline	1 µg/mL	Y	N
	10 µg/mL	Y	N
	100 µg/mL	Y	N
Moxifloxacin	1 µg/mL	Y	N
	10 µg/mL	Y	N
	100 µg/mL	Y	N
	1 mg/mL	Y	N
	2 mg/mL	Y	N
	4 mg/mL	Y	Y
	6 mg/mL	Y	Y
	10 mg/mL	Y	Y
	100 mg/mL	N	N
Metronidazole	1 µg/mL	Y	N
	10 µg/mL	Y	N
	100 µg/mL	Y	N
	1 mg/mL	Y	N
	2 mg/mL	Y	N
	4 mg/mL	Y	N
	6 mg/mL	N	Y
	10 mg/mL	N	Y
**Voriconazole**	10 µM	Y	N
	100 µM	Y	N
	500 µM	Y	N
	750 µM	Y	N
	**1 mM**	**N**	**N**
	1.25 mM	N	alive, but clumping
	1.5 mM	N	alive, but clumping
	2 mM	N	Y

Table S3. Primers used in qPCR and cloning

qPCR target	Control plasmid	Primer name	Primer Sequence (5’-3’)
Cancer-N1N2	pCR-SteamerLTR-N1N2	ClamLTR-F3<br>N1N2can-R3	TTCAATCATTCAACGCATAACC<br>TCGCTGAGAATTTTTCGGTGT
Total-N1N2	pCR-SteamerLTR-N1N2	N1N2-F3<br>N1N2-R1	CCCAGGGCAAGAGGAATATGGT<br>GGATACTGCAAGCTTCTTGGAA
Cancer-HL03	pIMHL03c2-EF1α	ClamLTRF2<br>IMHLO3c2-R2	ACATGCACATTAAAAGTTATCG<br>TCTGGGTCATGAATAACGTCA
EF1α	pIMHL03c2-EF1α	ClamEF1-F3<br>ClamEF1-R2	GGGAAAAGAGGGCAAGGTGAC<br>TTTCTTCTTCCCACCGACTGC
Cloning primers			
	pCR-SteamerLTR-N1N2	ClamLTR-F2<br>98171_conR1	ACATGCACATTAAAAGTTATCG<br>GGATACTGCAAGCTTCTTGGAA
	pIMHL03c2-EF1α	ClamLTR-F2[1]<br>ClamLTR-R1[1]<br>ClamA-EF1aFor[2]<br>ClamNS-EF1aRev[2]	ACATGCACATTAAAAGTTATCG<br>TTAGTATAGCCAATACTGTTAC<br>tagggcccGAAGGATGAGGGAAAAGAGGG<br>atGCGGCCGCatcctgcaggCACCTTTTCCTGCTATGGTGC

1 Cloning of the *Steamer* fragment to generate pIMHL03c2 was done via inverse PCR, as described in Arriagada et al. 2014.

2 Primers were used to amplify *EF1α* from genomic DNA, the product was cut with ApaI and NotI (NEB), and ligated into pIMHL03c2.
