# Supplementary figures and images for: Survival and Detection of Bivalve Transmissible Neoplasia from the Soft-Shell Clam Mya arenaria (MarBTN) in Seawater
Source: Pathogens. 2022 Feb 23;11(3):283. doi: 10.3390/pathogens11030283 (PMC8955499; doi:10.3390/pathogens11030283)

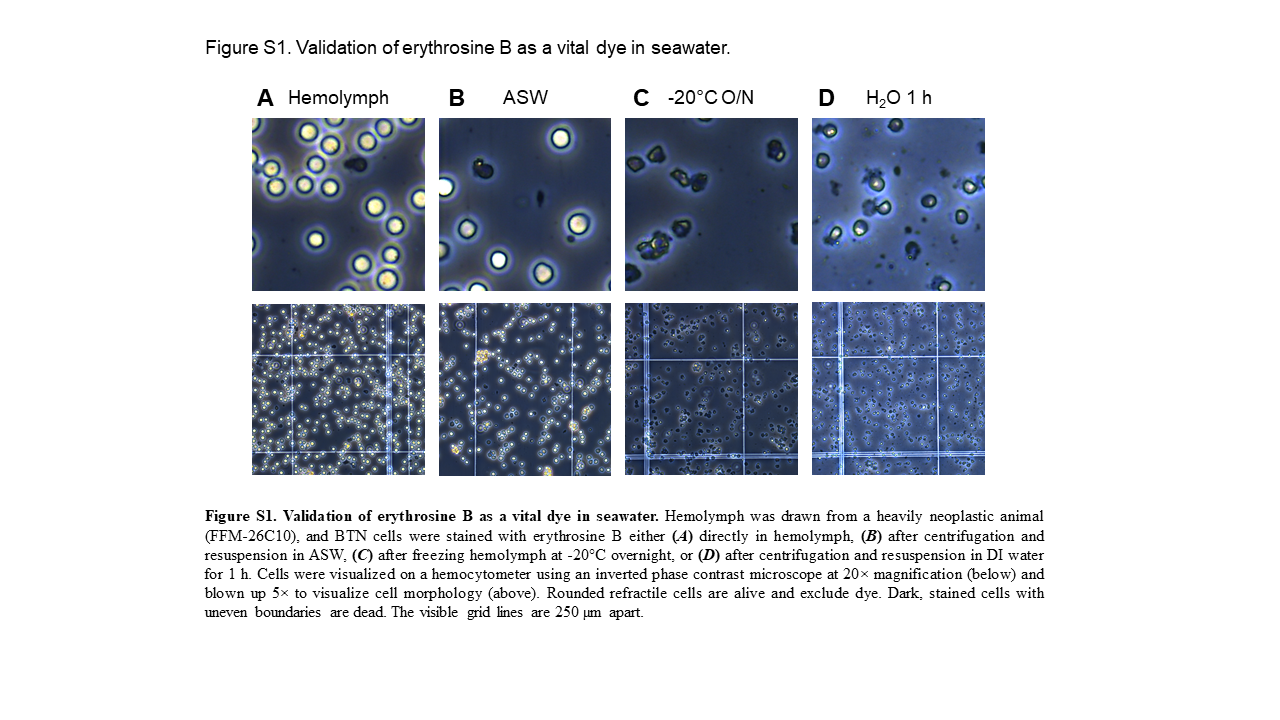

Supplement: Supplementary file 1 [file pathogens-11-00283-s001.zip › FIgureS1-2022-02-15.tif]

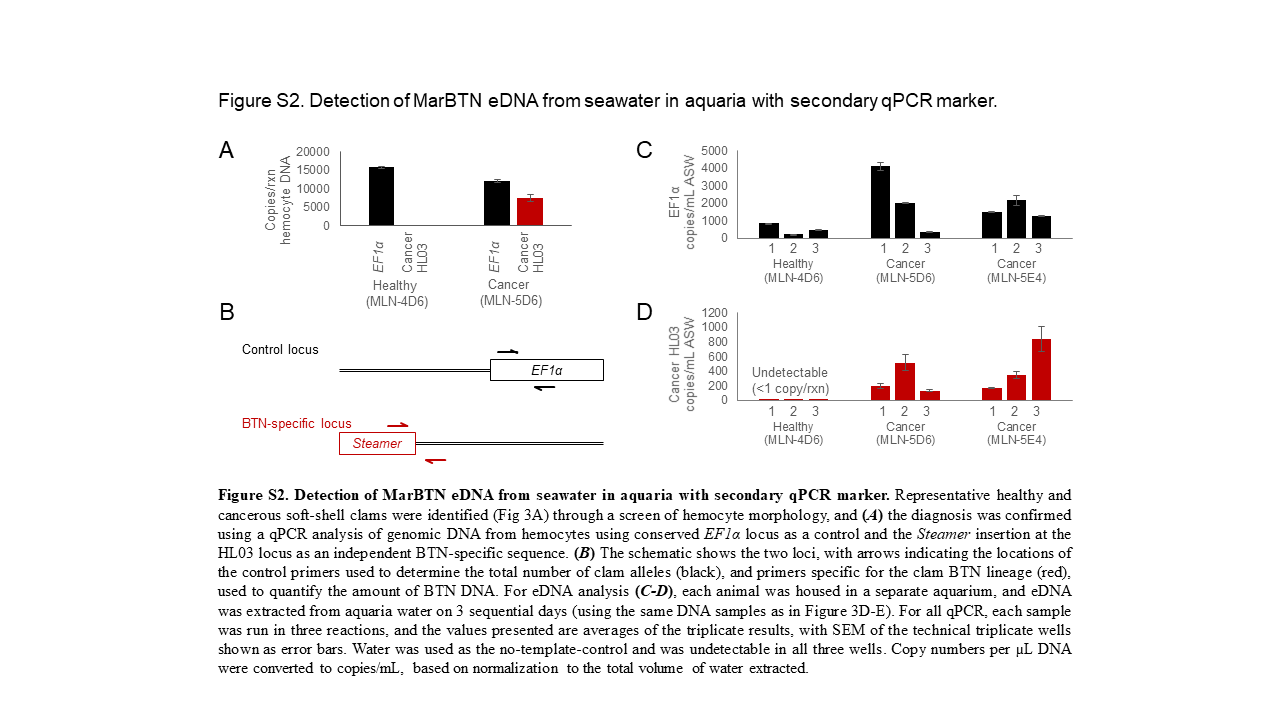

Supplement: Supplementary file 1 [file pathogens-11-00283-s001.zip › FigureS2-2022-02-15.tif]

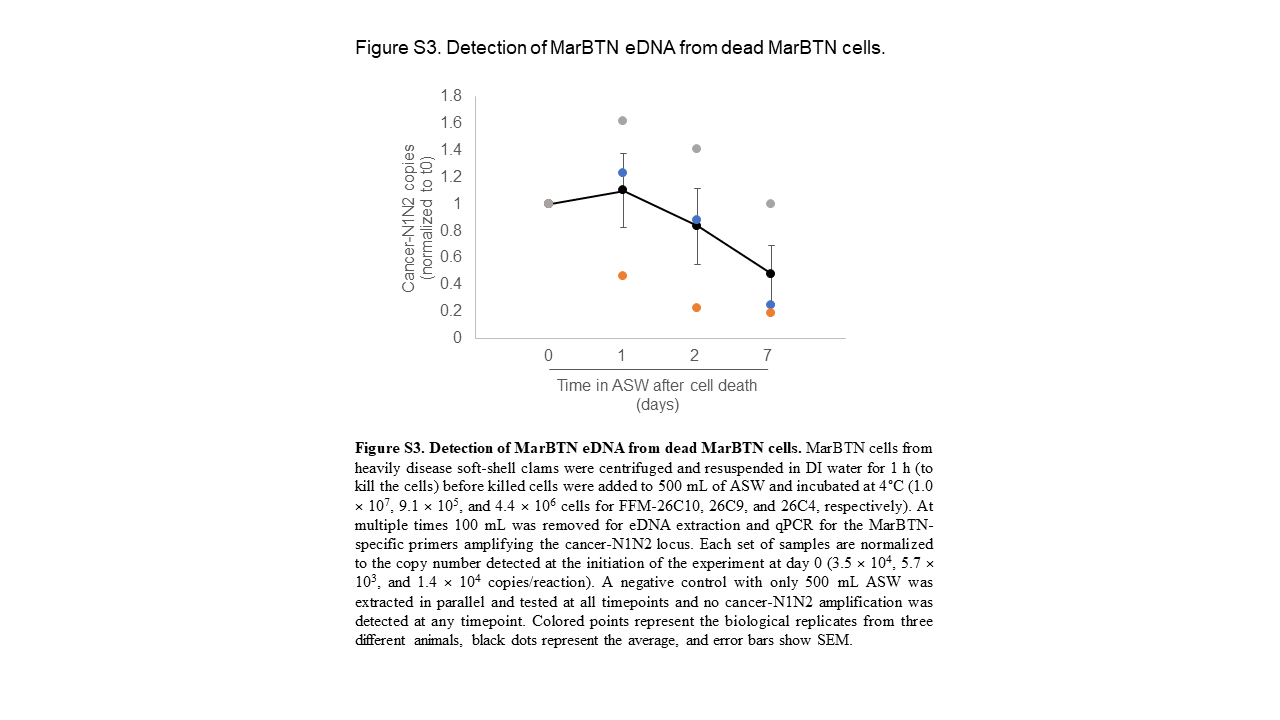

Supplement: Supplementary file 1 [file pathogens-11-00283-s001.zip › FigureS3-2022-02-15.tif]
